# *MDFI* is a novel biomarker for poor prognosis in LUAD

**DOI:** 10.3389/fonc.2022.1005962

**Published:** 2022-10-10

**Authors:** Pengyu Chen, Zhen Quan, Xueyu Song, Zhaojia Gao, Kai Yuan

**Affiliations:** ^1^ Division of Thoracic Surgery, The Affiliated Changzhou Second People’s Hospital of Nanjing Medical University, Changzhou, China; ^2^ School of Medicine, Dalian Medical University, Dalian, China; ^3^ Heart and Lung Disease Laboratory, The Affiliated Changzhou Second People’s Hospital of Nanjing Medical University, Changzhou, China

**Keywords:** *MDFI*, LUAD, TCGA, high expression, prognosis

## Abstract

**Background:**

Approximately 80% of lung cancers are non-small cell lung cancers (NSCLC). Lung adenocarcinoma (LUAD) is the main subtype of NSCLC. The incidence and mortality of lung cancer are also increasing yearly. Myogenic differentiation family inhibitor (*MDFI*) as a transcription factor, its role in lung cancer has not yet been clarified.

**Methods:**

LUAD data were downloaded from The Cancer Genome Atlas (TCGA) database and Gene Expression Omnibus (GEO), analyzed and plotted using the R language. Associations between Clinical information and *MDFI* expression were assessed using logistic regression analyses to explore the effects of *MDFI* on LUAD. Two sets of tissue microarrays (TMAs) further confirmed the overexpression of *MDFI* in LUAD and its impact on prognosis. In addition, we examined the correlation between *MDFI* and immune infiltration. To investigate the effect of *MDFI* on the biological behavior of LUAD tumor cells by GSEA and GO/KEGG analysis. The survival status and somatic mutational characteristics of patients according to *MDFI* levels were depicted and analyzed.

**Results:**

Expression of high *MDFI* in LUAD tissues *via* analyzing TCGA dataset (*P <*0.001). Kaplan-Meier survival analysis indicated a poor prognosis for those patients with LUAD who had upregulated *MDFI* expression levels (*P <*0.001). This was also verified by two groups of TMAs (*P*=0.024). Using logistic statistics analysis, *MDFI* was identified as an independent predictive factor and was associated with poor prognosis in LUAD (*P <*0.001, *P* =0.021). Assessment of clinical characteristics, tumor mutation burden (TMB), and tumor microenvironment (TME) between high- and low-expression score groups showed lower TMB, richer immune cell infiltration, and better prognosis in the low-risk group.

**Conclusion:**

This study showed that *MDFI* was overexpressed in LUAD and was significantly associated with poor prognosis, indicating that *MDFI* may be used as a potential novel biomarker for the diagnosis and prognosis of LUAD. *MDFI* is associated with immune infiltration of LUAD and it is reasonable to speculate that it plays an important role in tumor proliferation and spread. In view of the significant differences in *MDFI* expression between different biological activities, LUAD patients with *MDFI* overexpression may obtain more precise treatment strategies in the clinic.

## Introduction

Lung cancer is one of the most common malignant tumors in the world, and is the malignant tumor with the highest mortality rate in the world. NSCLC accounts for approximately 80% of all lung cancers. Its cancer cells grow and divide slowly and spread and metastasize relatively late. About 75% of patients are in the middle and advanced stages, and the 5-year survival rate is very low (1). LUAD is the most common subtype of NSCLC (2), accounting for 50% of all lung cancer diagnoses, and its frequency is increasing (3, 4). Among all NSCLC subtypes, LUAD is the most heterogeneous and aggressive, and has a very high tumor mutational burden associated with *EGFR*, *KRAS*, *BRAF*, *ERBB2*, *TP53*, *ALK*, *STK11*, and TTE1 mutations (5, 6). In the past 50 years, China has reported a significant increase in the incidence and mortality of lung cancer. The incidence and mortality of male lung cancer rank first among all malignant tumors in China, and the incidence of female lung cancer ranks second and the mortality rate ranks second (7). However, 5 years survival for patients with stage I NSCLC is roughly 80%, and patients with stage II to stage III disease have a 5 years survival of 13–60% (8). Although the addition of adjuvant chemotherapy in patients with a specific stage can increase survival by 5-10%, there is a significant amount of toxicity associated with it (9). The opportunity to improve survival is evident in early-stage disease and is driving research to integrate targeted therapies and ICIs (1). There is space for improvement in the treatment of LUAD and scientists delve into the identification of molecular markers associated with tumors and combine them with pathological classifications that affect personalized treatment of patients. In order to highly accurately predict patient survival and/or response to individualized treatment, new biomarkers were identified in those with LUAD.

By excavating the TCGA database, we found that *MDFI* may be a novel lung cancer–related candidate target. *MDFI* is a Protein Coding gene. Diseases associated with *MDFI* include Erythema Infectiosum and Viral Exanthem (10). This protein is a transcription factor that negatively regulates other myogenic family proteins (11). *MDFI* is overexpressed in breast, colorectal, and liver cancers and may promote tumorigenesis in certain tissues (12–14). However, the role of *MDFI* in lung cancer has not yet been reported. We attempted to elaborate the prognostic value of *MDFI* in LUAD by exploring TCGA database in this study.

## Methods and materials

### Patient data acquisition

We searched the GEO database for high-throughput sequencing or microarray data on LUAD and selected 4 LUAD transcriptome datasets with different sample sizes: GSE43458, GSE62948, GSE116959, GSE139032 (15–18). All of these datasets including lung tumors and para-tumor tissues. We searched the TCGA database (19) and obtained patient data for the LUAD cohort based on legitimate research objectives (20). The TCGA-LUAD cohort contained a total of 599 participants, including 59 normal patients and 539 patients with lung adenocarcinoma tumors. This includes mRNA data, clinicopathological data, and somatic mutation data. Some patients with missing data were excluded, and 535 oncology patients were enrolled in the study (21).

### Tissue microarray and analysis of immunohistochemical results

To further evaluate the expression of *MDFI* in NSCLC, we obtained a tissue microarray (TMA) from Superbiotek (Shanghai, China). It containing 60 pairs of NSCLC specimens and para-tumor tissues. In addition, we constructed a TMA containing 140 NSCLC tissues [included LUSC (n = 80), LUAD (n = 51), adenosquamous carcinoma (n = 5), Bronchioloalveolar carcinoma (n = 3), and sarcomatoid carcinoma (n = 1)] and 10 normal lung tissues. The reasonable tumor stage of these patients was determined based on the World Health Organization criteria and the International TNM classification (22). The patient did not receive radiotherapy or chemotherapy, and did not have other tumors within 5 years before surgery (**Supplementary Table 1**). Two groups of TMAs were deparaffinized with a conventional protocol and rehydrated according to a standard protocol for immunohistological (IHC) examination. Primary antibodies *MDFI* goat antibody (1:500, ProSci, PSI-42-165) were used (23, 24).

Then analyzing the staining results by Image Pro Plus 6 software (IPP6), the staining area (Area) and integrated optical density (IOD) can be obtained. The mean optical density (MOD) can be obtained by taking the ratio of the two, and the MOD value is positively correlated with the staining intensity of the tissue. By analyzing the MOD of *MDFI* in NSCLC, para tumor and normal tissues can be further studied. We mapped the immunostaining heat map of tumor tissue and normal lung tissue adjacent to the tumor, and labeled the corresponding expression scores. In addition, we also compared the expression levels of *MDFI* in the tumor and normal groups in 4 GEO validation sets.

### Gene expression and survival time

High-volume data downloaded from the TCGA was managed using the R programming language (25). The results of unpaired and paired samples were analyzed by independent and paired sample t-test, respectively (26). We have selected several groups of characteristics with more obvious differences for comparative display. Boxplots plots, using Age, Pathologic stage, T stage, Smoker and so on as the variable, were generated to calculate differential expression of *MDFI*. Differences in global gene expression levels between the normal tissues and tumor tissues of patients with LUAD were analyzed with an R package and *P* < 0.05. Kaplan‐Meier analysis was used to evaluate the prognostic value of *MDFI* in LUAD patients (25).

### Immune cell and immune function analysis

The gene set analysis of variance (GSVA) procedure of the ‘GSVA’ and ‘GSEA Base’ package of R software was used to calculate gene signature enrichment scores (GSVA scores) for immune cells and immune function in each sample, which is called relative immune cell abundance [10.1038/ng765.] (27, 28). Heat maps of immune cells and immune function within disease groups and normal groups have been established using the “pheat map” software package. We then performed a correlative analysis of immune cells and immune function separately using the corresponding package, which was calculated using the Pearson correlation method (29). The scores of immune cells and immune function were also compared in the *MDFI* high expression group and low expression group (30).

### Functional enrichment analysis

First, we divided the TCGA-LUAD cohort patients into two groups with high and low *MDFI* expression levels according to the *MDFI* expression. Differential analysis between groups was performed using the ‘limma’ package of R software (31), and differential genes were screened according to the criteria of adj. *P <*0.05, |log FC| >0.5. To explore the functions of the differentially expressed genes in *MDFI*, the screened genes were enriched for Gene Ontology (32) and Kyoto Encyclopedia of Genes and Genomes (33) pathway terms, respectively (34). We used the “cluster Profiler” package in R software to carry out the analysis and plotted the enriched pathway results separately (35).

### Somatic mutation analysis

We downloaded mutation data from TCGA for the LUAD patient cohort. To investigate the relationship between *MDFI* and mutations, we first divided the mutation profile of patients into high *MDFI* mutation group and low *MDFI* mutation group depending on the expression of *MDFI*. We assessed the mutations in the two groups separately using the R software “Maftools”, and plotted the waterfall of mutations (36). We analyzed differences in mutations in different *MDFI* expression groups, as well as the differences in the number and location of mutations in the same mutated gene due to different *MDFI* expression (37).

### Predicted drugs

Finally, we used protein-drug interaction data from the DSigDB database (38) to identify potential drugs that could benefit lung adenocarcinoma. We adopted the following tests: t-test or wilcoxon test for differences between groups depending on the data, and pearson or spearman method for correlation analysis. Survival analysis was performed using Kaplan-Meier and Cox regression analysis, and the results were evaluated using LogRank test, respectively (25).

### Statistical analysis

R software (version 4.1.1; The R Foundation for Statistical Computing) was used for all statistics of this article. All statistical tests were double-sided, and *P* values less than 0.05 were evaluated as significant (28).

## Result

### *MDFI* expression in TCGA dataset

**Figure 1:**
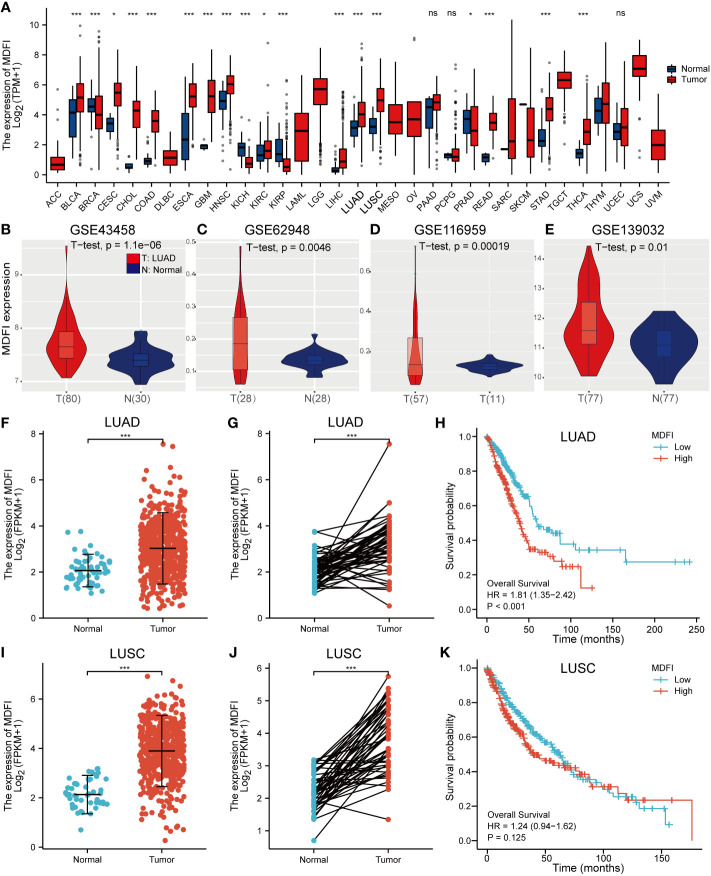
Analysis of MDFI expression in tumors, **(A)** Pan-cancer analysis of MDFI, **(B–E)** Expression analysis of MDFI in 4 sets of GEO datasets, **(F)** Expression analysis of MDFI in unpaired samples of LUAD, **(G)** MDFI in the LUAD expression analysis in paired sample, **(H)** Prognostic analysis of the survival of MDFI in LUAD, **(I–K)** Expression analysis of MDFI in LUSC. **(A, F–K)** Data from TCGA database. *p < 0.05, ***p < 0.001. ns, no significance.

Significantly increased levels of *MDFI* expression in LUAD and LUSC compared to normal lung tissue by pan-cancer analysis using TGCA data (*P <*0.001) (**Figure 1A**). Likewise, high expression of *MDFI* in LUAD and low expression in normal lung tissue was verified in four GEO validation sets (**Figures 1B–E**). However, in terms of Kaplan-Meier curve, LUAD with high *MDFI* expression has a significantly worse prognosis (*P* < 0.001), while the expression of *MDFI* was not significant with the prognosis of LUSC (*P* = 0.125). Therefore, the study focused on the role of *MDFI* in LUAD (**Figures 1F–K**).

### Characteristics of patients

A total of 535 LUAD patients with the required clinical features were acquired from TCGA data portal in July 2022 (**Table 1**). Among them, 249 were male (46.5%) and 286 were female (53.5%). The median age of all patients was 66 years. In terms of LUAD pathologic stage, 294 patients were stage I (55.8%), 123 patients were stage II (23.3%), 84 patients were stage III (15.9%), and 26 patients were stage IV (4.9%). Subjects included 406 White (86.8%) and 62 non-White (13.2%). In terms of primary treatment outcome, PD + SD were 108 (24.2%), PR+CR were 338 (75.8%).

**Table 1:** Baseline Table of 535 NSCLC Patients in TCGA Database.

Characteristic	levels	Overall
n		535
Gender, n (%)	Female	286 (53.5%)
	Male	249 (46.5%)
Age, n (%)	<=65	255 (49.4%)
	>65	261 (50.6%)
Race, n (%)	Asian	7 (1.5%)
	Black or African American	55 (11.8%)
	White	406 (86.8%)
Smoker, n (%)	No	75 (14.4%)
	Yes	446 (85.6%)
Pathologic stage, n (%)	Stage I	294 (55.8%)
	Stage II	123 (23.3%)
	Stage III	84 (15.9%)
	Stage IV	26 (4.9%)
T stage, n (%)	T1	175 (32.9%)
	T2	289 (54.3%)
	T3	49 (9.2%)
	T4	19 (3.6%)
N stage, n (%)	N0	348 (67.1%)
	N1	95 (18.3%)
	N2	74 (14.3%)
	N3	2 (0.4%)
M stage, n (%)	M0	361 (93.5%)
	M1	25 (6.5%)
Primary therapy outcome, n (%)	PD	71 (15.9%)
	SD	37 (8.3%)
	PR	6 (1.3%)
	CR	332 (74.4%)
Anatomic neoplasm subdivision, n (%)	Left	205 (39.4%)
	Right	315 (60.6%)
Anatomic neoplasm subdivision2, n (%)	Central Lung	62 (32.8%)
	Peripheral Lung	127 (67.2%)
OS event, n (%)	Alive	343 (64.1%)
	Dead	192 (35.9%)
DSS event, n (%)	Alive	379 (76%)
	Dead	120 (24%)
PFI event, n (%)	Alive	309 (57.8%)
	Dead	226 (42.2%)
Age, median (IQR)		66 (59, 72)
number_pack_years_smoked, median (IQR)		37 (20, 50)

PFI, Progression Free Interval; OS, Overall Survival; DSS, Disease-Specific Survival; PD, progressive disease; SD, stable disease; PR, partial response; CR, complete Response.

### Immunohistochemical analysis

**Figure 2:**
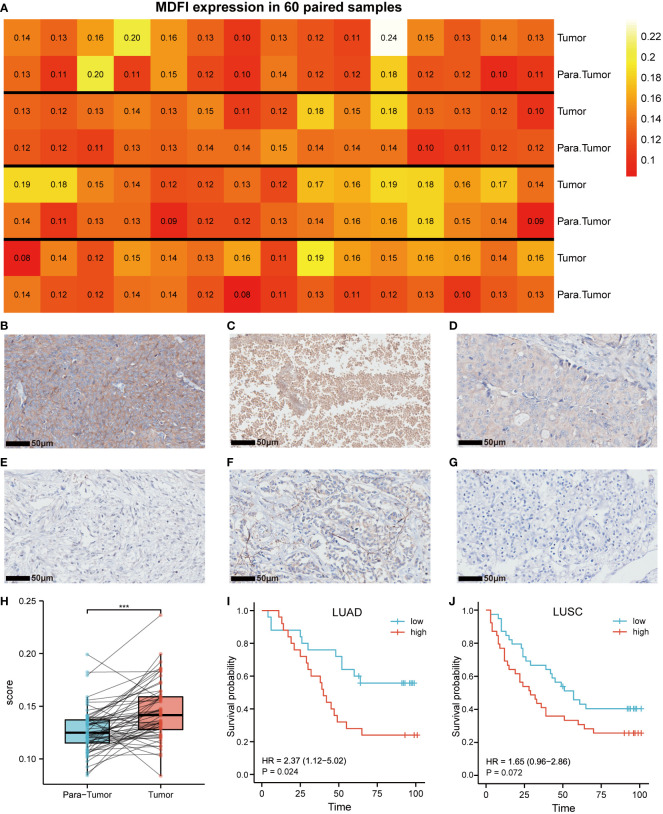
Expression of MDFI in tumor tissues. **(A)** Heat map of MDFI expression in 60 pairs of samples, **(B)** High expression of MDFI in lung cancer tissues, **(C)** MDFI is moderately expressed in lung cancer tissues, **(D)** Low expression of MDFI in lung cancer tissues, **(E)** Unstained normal tissue, **(F)** MDFI staining in invasive breast cancer tissue, **(G)** MDFI staining in clear cell renal cell carcinoma tissue, **(H)** Box plot of MDFI expression in 60 pairs of paired samples, **(I)** Prognastic analysis of MDFI in LUAD specimens, **(J)** prognostic analysis of MDFI in LUSC specimens, ***p < 0.001.

*MDFI*-specific antibody staining was performed on the two groups of TMAs constructed in advance, we could see various staining situations and plot 60 pairs of tumor and para-tumor immunostaining heatmap (**Figures 2A–G**). Meanwhile, we selected one column each of invasive breast cancer and clear cell renal cell carcinoma as the reference sample for the same staining procedure (**Figures 2F–G**). The staining results were processed by IPP6 software to obtain MOD values. By MOD analysis of 60 pairs of tumor and para-tumor, the results suggested that the expression of *MDFI* in tumors was indeed higher than that in normal tissues (*P* < 0.001) (**Figure 2H**). Further, the K-M curve suggested that high-expressing *MDFI* had a worse prognosis in LUAD (n = 51, *P* = 0.024), whereas the difference was not significant in LUSC (n = 80, *P* = 0.072) (**Figures 2I–J**). Our experimental results similarly corroborate previous results predicted by the TCGA database. High *MDFI* expression can be used as an independent prognostic marker in LUAD.

### Correlation between *MDFI* expression and clinical features

**Figure 3:**
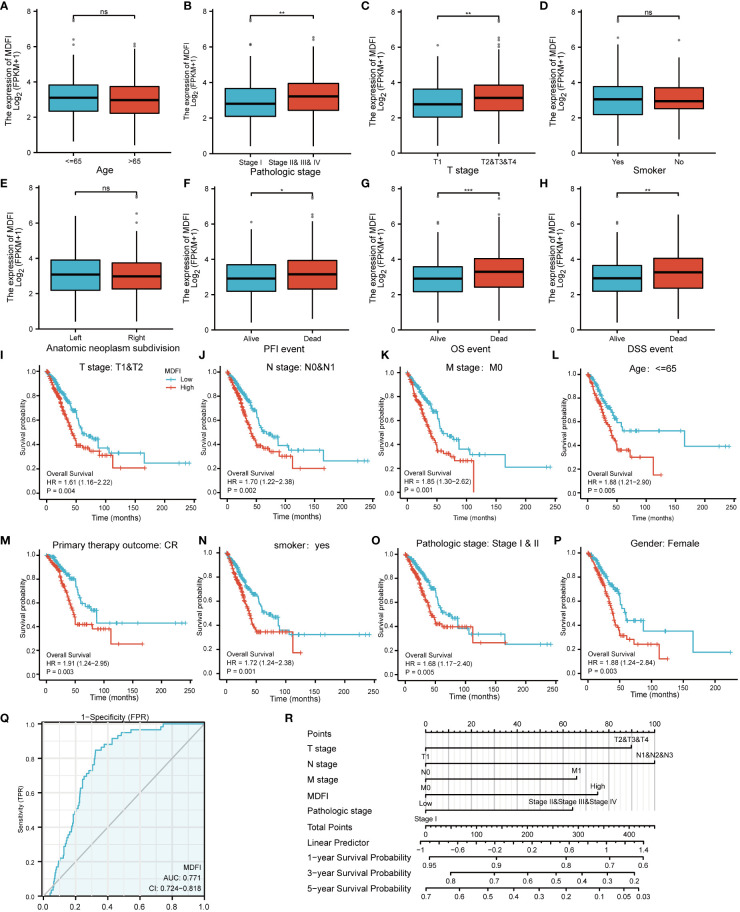
Association of MDFI expression with different LUAD characteristics and prognosis, date from TCGA, **(A–H)** Expression of MDFI in relation to different characteristics of LUAD, **(I–P)** Association between high MDFI expression in prognosis of LUAD with different characteristics, **(Q)** MDFI’s ROC curve analysis at LUAD, **(R)** Construction of a nomogram to assess the prognostic value of MDFI in LUAD. *p < 0.05, **p < 0.01, ***p < 0.001, ns, p > 0.05. PFI, Progression Free Interval; OS, Overall Survival; DSS, Diseases-Specific Survival; CR, complete response.

In LUAD patients, the relationship between *MDFI* and clinical characteristics is summarized in **Table 2**. Logistic regression analysis showed that *MDFI* gene expression is a categorical dependent variable associated with poor prognostic clinical features. High expression of *MDFI* was significantly associated with T stage (*P* =0.013), N stage (*P* =0.004), Pathologic Stage (*P <*0.001) and Primary therapy outcome (*P* =0.025) (**Table 2**). High expression of *MDFI* was significantly correlated with clinical stage (*P <*0.001), T stage (*P <*0.001), PFI event (*P <*0.05), OS event (*P <*0.001) and DSS event (*P <*0.01) (**Figures 3A–H**).

**Table 2:** Logistic analysis of the correlation between *MDFI* expression and clinical characteristics.

Characteristics	Total (N)	Odds Ratio (OR)	*P* value
T stage (T2&T3&T4 vs. T1)	532	0.630 (0.436-0.906)	0.013
N stage (N1&N2&N3 vs. N0)	519	0.582 (0.401-0.843)	0.004
M stage (M1 vs. M0)	386	0.685 (0.291-1.550)	0.370
Pathologic stage (Stage II&Stage III&Stage IV vs. Stage I)	527	0.540 (0.381-0.764)	<0.001
Gender (Male vs. Female)	535	1.262 (0.898-1.775)	0.180
Age (>65 vs. <=65)	516	1.205 (0.853-1.704)	0.289
Residual tumor (R2&R1 vs. R0)	372	0.331 (0.092-0.956)	0.057
Anatomic neoplasm subdivision (Right vs. Left)	520	1.209 (0.851-1.721)	0.290
number_pack_years_smoked (>=40 vs. <40)	369	0.762 (0.506-1.147)	0.193
Smoker (Yes vs. No)	521	0.807 (0.492-1.317)	0.393
Primary therapy outcome (PR&CR vs. PD&SD)	446	1.653 (1.069-2.573)	0.025
Anatomic neoplasm subdivision2 (Peripheral Lung vs. Central Lung)	189	1.156 (0.629-2.131)	0.640
Race (White vs. Asian&Black or African American)	468	1.088 (0.637-1.863)	0.757

PD, progressive disease; SD, stable disease; PR, partial response; CR, complete response.

### High expression of *MDFI* is an independent risk factor foroverall survival

High *MDFI* expression was associated with poor prognosis, as shown in Kaplan-Meier survival analysis (**Figures 3I–P**). Analysis of different clinical characteristics showed that high *MDFI* expression was significantly associated with poor prognosis in LUAD patients with T1&T2 stage (p=0.004), N0&N1 stage (*P* =0.002), M0 stage (*P* =0.001), less than 65 years (*P* =0.005), Primary therapy outcome: CR (*P* =0.003), smoker (*P* =0.001), pathologic stage I&II (*P* =0.005) and female (*P* =0.003) (**Figures 3B–I**). Univariate Cox analysis demonstrated that high *MDFI* expression was significantly correlated with poor overall survival (*P <*0.001). Multivariate Cox analysis confirmed MDIF gene expression was an independent risk factor for overall survival in patients with LUAD (*P* =0.021) (**Table 3**).

**Table 3:** Univariate and multivariate Cox regression analyses of clinical characteristics associated with overall survival.

Characteristics	Total(N)	Univariate analysis		Multivariate analysis	
		Hazard ratio (95% CI)	*P* value	Hazard ratio (95% CI)	*P* value
T stage (T3&T4 vs. T1&T2)	523	2.317 (1.591-3.375)	**<0.001**	1.589 (0.878-2.875)	0.126
N stage (N2&N3 vs. N0&N1)	510	2.321 (1.631-3.303)	**<0.001**	1.934 (0.800-4.671)	0.143
M stage (M1 vs. M0)	377	2.136 (1.248-3.653)	**0.006**	1.593 (0.597-4.248)	0.352
Pathologic stage (Stage III&Stage IV vs. Stage I&Stage II)	518	2.664 (1.960-3.621)	**<0.001**	1.111 (0.440-2.804)	0.824
Gender (Male vs. Female)	526	1.070 (0.803-1.426)	0.642		
Age (>65 vs. <=65)	516	1.223 (0.916-1.635)	0.172		
Race (Asian&Black or African American vs. White)	468	0.678 (0.415-1.109)	0.121		
Smoker (Yes vs. No)	512	0.894 (0.592-1.348)	0.591		
Anatomic neoplasm subdivision (Right vs. Left)	512	1.037 (0.770-1.397)	0.810		
Anatomic neoplasm subdivision2 (Peripheral Lung vs. Central Lung)	182	0.913 (0.570-1.463)	0.706		
Primary therapy outcome (PR&CR vs. PD&SD)	439	0.377 (0.268-0.530)	**<0.001**	0.345 (0.227-0.525)	**<0.001**
MDFI (High vs. Low)	526	1.892 (1.408-2.542)	**<0.001**	1.606 (1.075-2.398)	**0.021**

PD, progressive disease; SD, stable disease; PR, partial response;, CR, complete response; P, <0.05.

P values in bold in Table 3 represent P <0.05.

### Diagnostic value of *MDFI* expression in LUAD

We performed ROC curve analysis of *MDFI* gene expression data to evaluate the diagnostic value of this gene. The AUC area was 0.771 (CI =0.724-0.818). These results indicate that *MDFI* expression has certain value in the diagnosis of LUAD. A nomogram was constructed to predict the 1-, 3-, and 5-year survival probability of patients in combination with the expression level of *MDFI*, TNM stage, and pathological stage (**Figures 3Q, R**).

### Immune infiltration by *MDFI*

**Figure 4:**
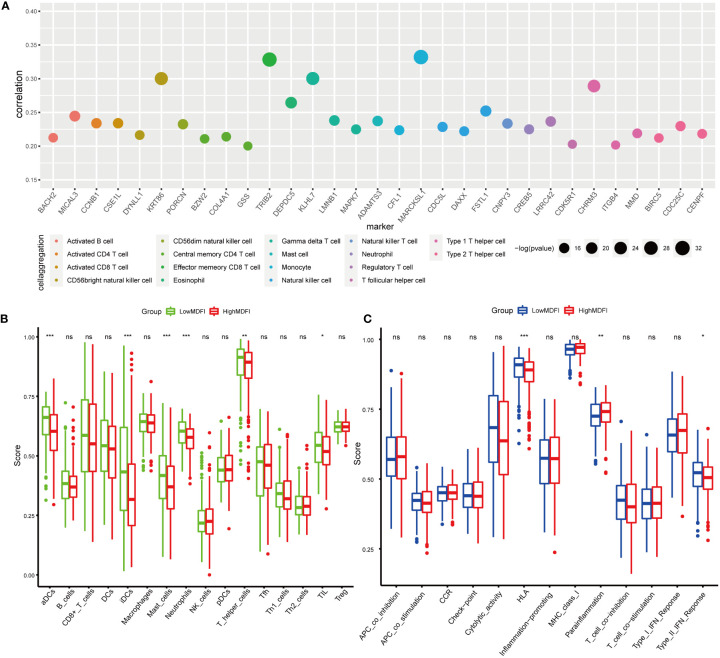
Immuno-infiltration analysis of MDFI in LUAD, data from TCGA, **(A)** Maker genes of immune cells associated with MDFI, **(B)** Relationship between the expression profile of MDFI and immune cells, **(C)** Relationship between the expression profile of MDFI and immune function. *p < 0.05, **p < 0.01, ***p < 0.001, ns, p > 0.05.

To investigate the relationship between *MDFI*-associated immune cells and immune function, we screened the immune cell marker genes associated with the *MDFI* high expression group at *P <*0.05 (**Figure 4A**). We found that high *MDFI* expression was associated with most immune cells, with significant marker genes for Effector memory CD8 T cells and Monocyte, such as *TRIB2* and *MARCKSL1*.

Immuno-infiltration analysis was then performed in the *MDFI* high and low expression groups, in which aDCs (*P* < 0.001), iDCs (*P* < 0.001), Mast cells (*P* < 0.001), Neutrophils (*P* < 0.001), T helper cells (*P* < 0.01) and TIL (*P* < 0.05) showed differential expression (**Figure 4B**). In terms of immune function, HLA (*P* < 0.001), Para-inflammation (*P* < 0.01) and Type II IFN Reponse (*P* < 0.05) were significant (**Figure 4C**).

### Enrichment analysis of *MDFI*-related genes

**Figure 5:**
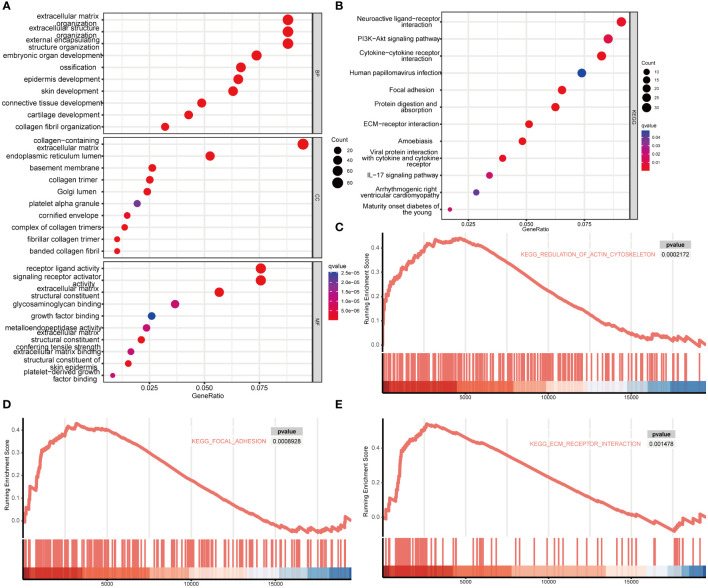
Enrichment analysis of MDFI in LUAD, data from TCGA. **(A)** GO. BP/CC/MF enrichment analysis, **(B)** KEGG enrichment analysis, **(C–E)** GSEA enrichment analysis.

To investigate the potential role of *MDFI* in LUAD, GO analysis and KEGG enrichment were used to analyze the function of *MDFI* differential genes. Among them, GO: BP/CC/MF showed 827 *MDFI* high expression group-related genes mainly acting in cornification, keratinization, cornified extracellular matrix and envelope structural constituent (**Figures 5A, B**). GSEA enrichment results were consistent with the direction of the above analysis, with up-regulated genes showing significant performance in regulation of actin cytoskeleton (*P <*0.001), focal adhesion (*P <*0.001) and extracellular matrix (ECM) receptor interaction (*P* =0.001) (**Figures 5C–E**).

### *MDFI*-related gene co-expression analysis

**Figure 6:**
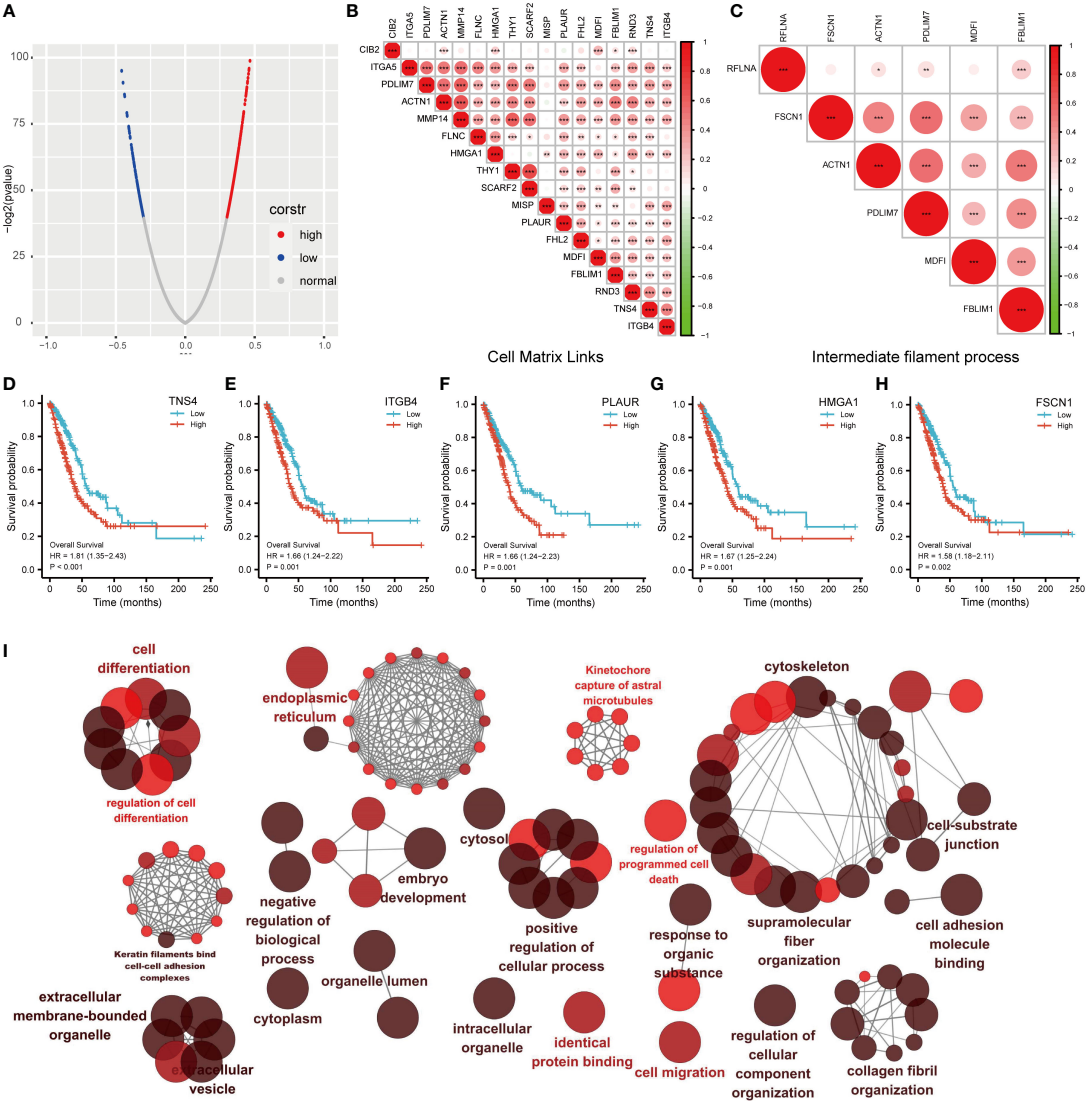
Correlation analysis of MDFI in LUAD **(A)** MDFI-related genes in LUAD, **(B)** Heat map of MDFI-related genes in Cell Matrix Links, **(C)** Heat map of MDFI-related genes in intermediate filament process, **(D–H)** Prognostic analysis of some MDFI-related genes in LUAD, **(I)** Visualization of the interaction network of genes strongly associated with MDFI by Cytoscape (ClueGo module). Node size indicates the mapped gene number; the node color schedule represents the p value, the darker the color, the smaller the p-value. **(A–H)** Data from TCGA. *p < 0.05, **p <0.01, ***p < 0.001.

We performed co-expression analysis of genes closely related to *MDFI* expression to characterize genes associated with *MDFI* expression in the LUAD cohort (**Figure 6A**). Further detailed analysis of the association of cellular Matrix Links and intermediate filament bioactivity-related genes with their *MDFI* revealed several prominent genes, such as *TNS4*, *ITGB4*, *PL AUR*, *HMGA1*, and *FSCN1*. Most of these genes are positively correlated with *MDFI* expression, and these genes have an impact on both LUAD and prognosis (**Figures 6B–H**). Next, the interaction networks of these co-expressed genes were visually analyzed to embody the association of these genes and biological functions. The network of these gene interactions suggests that the significantly associated biological functions are Cell differentiation, cytoskeleton, regulation of cell differentiation and positive regulation of cellular process (**Figure 6I**).

### Relationship between somatic mutations and *MDFI* expression in LUAD

To investigate the critical role of *MDFI* in tumor progression and tumor cell dissemination and metastasis, we explored whether the distribution of mutations in the LUAD cohort was influenced by *MDFI* gene expression. We collected mutation profiles from the LUAD cohort in the TCGA database and plotted waterfalls according to MDIF expression. Interestingly, the top five mutated genes in the two groups of differential genes were the same *TP53* (57%; 42%), *TTN* (46%; 40%), *MUC16* (42%; 39%), *CSMD3* (42%; 38%) and *RYR2* (39%; 29%) (**Supplementary Figures A, B**).

**Figure 7:**
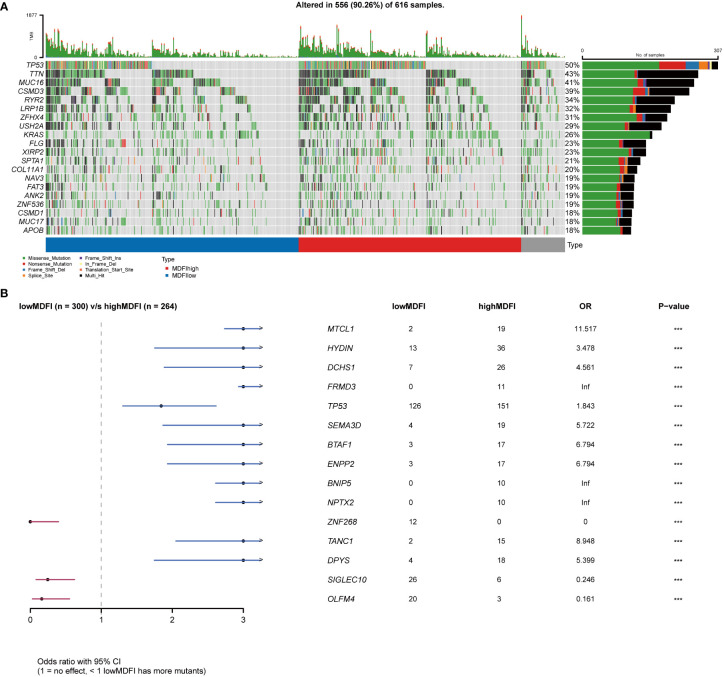
Relationship between MDFI and LUAD somatic mutation, data from TCGA. **(A)** Somatic mutation in MDFI-low and MDFI-high expression groups, **(B)** Comparison of mutation in MDFI high expression group and low expression group. ***p < 0.001.

Mutated genes were more frequent in the high-expression group, including *MTCL1*, *HYDIN*, *DCHS1*, *FRMD3*, *TP53*, *SEMA3D*, *BTAF1*, *ENPP2*, *BNIP5*, *NPTX2*, *TANC1* and *DPYS*, whereas the low-expression group included *ZNF268*, *SIGLEC10* and *OLFM4* (**Figure 7B**).

**Figure 8:**
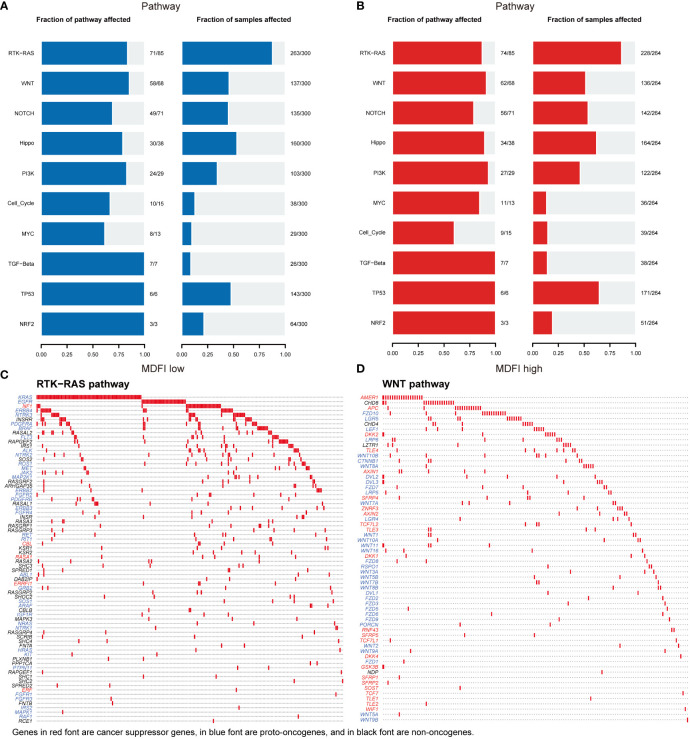
MDFI in LUAD involves mutations in the pathway data from TCGA **(A)** Pathway mutations of MDFI-low in LUAD, **(B)** Pathway mutations of MDFI-high in LUAD, **(C)** Mutations involved in RTK-RAS pathway, **(D)** Mutations involved in the WNT pathway.

Then we performed a visual analysis of mutations in the pathways involved in both sets of genes, including the number of genes mutated in the pathway and the number of samples mutated (**Figures 8A, B**). It can be seen that in terms of Pathway, the mutation in the *MDFI* low group was still lower than that in the *MDFI* high, and then the RTK-RAS pathway and WNT pathway of the first two pathways in the *MDFI* high group were separately plotted as waterfalls (**Figures 8C, D**).

### Predicted drugs

Bazedoxifene (adj. *P* =0.027), Pentadecafluorooctanoic acid (adj. *P* =0.0313), Hexachlorobiphenyl (adj. *P* =0.0313), Pentachlorphenyl (adj. *P* =0.0313), Arbutin (adj. *P* =0.0313), Kojic acid (adj. *P* =0.0313) and Nickel sulfate (adj. *P* =0.049371) were screened for potential drugs that may be beneficial in lung adenocarcinoma using protein-drug interaction data from the DSigDB database, and may be effective in LUAD patients with *MDFI* overexpression (**Table 4**).

**Table 4:** Predict potential drug.

Term	Overlap	*P*	adj. *P*	Combined Score
Bazedoxifene CTD 00004022	1/45	0.00225	0.027	121662.4978
Pentadecafluorooctanoic acid CTD 00001078	1/203	0.01015	0.0313	90874.00322
2,2’,4,4’,5,5’-Hexachlorobiphenyl CTD 00000731	1/208	0.0104	0.0313	90369.46872
3,3’,4,4’,5-Pentachlorobiphenyl CTD 00001077	1/237	0.01185	0.0313	87657.53732
arbutin CTD 00005438	1/282	0.0141	0.0313	84030.01469
kojic acid CTD 00000624	1/313	0.01565	0.0313	81844.61948
NICKEL SULFATE CTD 00001417	1/576	0.0288	0.049371	68904.42357

adj. P: adjusted P –value.

## Discussion

*MDFI* (Myogenic differentiation Family Inhibitor) is a Protein Coding gene (39). This protein is a transcription factor that negatively regulates other myogenic family proteins (11). It is highly expressed in a variety of cancer tissues, including Liver hepatocellular carcinoma, Breast invasive carcinoma, Colon adenocarcinoma/Rectum adenocarcinoma, etc (12–14). There is currently limited literature on the potential prognostic impact of *MDFI* in NSCLC. Therefore, we conducted a study on the potential role of *MDFI* in NSCLC, analyzing *MDFI* expression in NSCLC cohorts for the first time. In the context of TCGA data, we retrospectively analyzed 535 LUAD patients. The experimental results highlight that in LUAD patients, *MDFI* expression was significantly higher in tumors than in para-tumor. Similarly, it was validated in 4 GEO datasets and external immunohistochemistry experiments. Further, the expression levels of *MDFI* also differed in clinical stage, T stage, PFI event and OS event. We found that both RNA-seq data and TMA results showed that the expression level of *MDFI* was related to clinical prognosis. LUAD patients with high *MDFI* expression have a worse prognosis. Moreover, the K-M curves of female patients aged < 65 years with earlier tumor stage also supported the above results. Similarly, multivariate analysis showed that *MDFI* can be used as an independent prognostic factor in LUAD and is associated with poor prognosis in LUAD. Moreover, the AUC in our plotted ROC curve indicates that *MDFI* can be used as an indicator to predict LUAD.

The analysis of CIBERSORT showed that the expression level of *MDFI* was positively correlated with the infiltration level of most immune cells in LUAD, including CD4 T cells, Activated CD8 T cells, Effector memory CD8 T cells and Monocyte. The marker genes of these immune cells play an important role in tumor immune response, immune escape, proliferation, migration and invasion, promoting the progression of this LUAD. *TRIB2* and *MARCKSL1* are the most prominent marker genes in these immune cells. Critical role of *TRIB2* in cancer and drug resistance to therapy, *TRIB2* interacts with *MAPKK*, *AKT* and *NFkB* proteins and participates in cell survival, proliferation and immune responses (40, 41). Ectopic or intrinsic high expression of *TRIB2* induces drug resistance by promoting phosphorylation of *AKT* through its *COP1* structural domain. significantly increased expression of *TRIB2* in tumor tissue correlates with increased phosphorylation of *AKT*, *FOXO3a*, *MDM2* and impaired treatment response. This ultimately led to extremely poor clinical outcomes (42). When *MARCKSL1* phosphorylation is inhibited, actin mobility is increased, filamentous sodium formation is impaired, and laminar lipid formation is enhanced, as is cell migration, and we speculate that the same process may be going on in LUAD, thereby promoting tumor cell proliferation, migration, and invasion (43). *MARCKSL1* promoted the progression of lung adenocarcinoma by regulating epithelial–mesenchymal transition (EMT) (44). These results suggest that *MDFI* may play an important role in immune escape in the LUAD microenvironment (45). Moreover, it can be seen that the HLA presentation pathway is more pronounced in the low *MDFI* expression group, while tumor cells are more likely to evade immune detection in the absence of the HLA presentation pathway, thereby promoting tumor progression (46).

Keratin, the major intermediate filament protein of epithelial cells, and the cytoskeleton play multiple key roles in the cell, from cell migration to organelle dynamics (47, 48). They not only have a positive biological role in tumor progression and tumor cell dissemination and metastasis, but also often maintain their specific expression pattern during malignant transformation of cancer, so they are also widely used as tumor markers in cancer diagnosis. Both GO/KEGG enrichment and GSEA analysis indicate that *MDFI* has a prominent performance in the above biological processes. Keratins act as epithelial cell markers, which makes their role in cancer progression, diagnosis and treatment an important focus of research. Among them, keratin 1(K1) can act as a cell surface receptor in cancer, and KEGG enrichment analysis also shows that cytokine receptor action is more significant (49, 50); while keratin 17 (K17) plays a role in DNA damage response and tumor initiation. Moreover, keratin is an essential element of the cytoskeleton in normal and malignant epithelial cells (49, 51). Cancers often maintain their specific keratin expression pattern during malignant transformation, and therefore keratin is widely used as a tumor marker in cancer diagnosis. Keratin plays an active biological role in tumor cell dissemination and metastasis (52).

We speculate that detection and treatment for *MDFI* may allow earlier diagnosis of tumors and limit further tumor growth. Several genes that were highly correlated and positively correlated in the subsequent co-expression analysis of *MDFI* showed the same differential performance for the prognosis of LUAD, suggesting that low expression is better for the prognosis. It further strengthens the important role and predictive value of *MDFI* in the prognosis of LUAD. The network of these gene interactions suggests Cell differentiation, cytoskeleton, regulation of cell differentiation and positive regulation of cellular process that are considered to be important process processes of tumor progression (53). In the analysis of somatic mutations in the high and low groups, the proportion of the top five mutated genes in the low expression group was lower than that in the high expression group, and it was speculated that the tumor cells with high expression of *MDFI* produced a large number of DNA replication errors in the proliferation and spread, indicating a worse prognosis (54). We mapped the RTK-RAS pathway and the WNT pathway of the first two pathways in the high *MDFI* group as waterfalls, respectively, LUAD-derived Wnts increase the proliferation/stemness potential of cancer cells, and LUAD cells use paracrine Wnt1 signaling to induce immune resistance, which could provide a new pathway therapeutic option for LUAD with high *MDFI* expression (55–57). Finally, we predicted that Bazedoxifene performed most prominently in *MDFI*-related LUAD. Bazedoxifene, or combined with oxaliplatin, significantly induced apoptosis, inhibited cell viability, colony formation, and cell migration in colon cancer cells, and we speculated that it may have the same effect in LUAD (58).

The above data and experimental results all suggest that *MDFI* is a closely related gene in LUAD, but our study still has some limitations. Our exploration of the role of *MDFI* in LUAD is based on data already available in GEO and TCGA databases, coupled with external data collected for validation and the specimens are old, and there are individual unstained conditions. And, no *in vivo* and *in vitro* experiments were performed to further verify the role of *MDFI* in immune escape and proliferation and spread of tumors, which also points the direction for future work.

## Data availability statement

The datasets presented in this study can be found in online repositories. The names of the repository/repositories and accession number(s) can be found in the article/**Supplementary Material**.

## Ethics statement

The studies involving human participants were reviewed and approved by Research Ethics Committee of Changzhou Second People’s Hospital Affiliated to Nanjing Medical University. The patients/participants provided their written informed consent to participate in this study. Written informed consent was obtained from the individual(s) for the publication of any potentially identifiable images or data included in this article.

## Author contributions

KY designed the research. XS and ZG performed the research and collected the data. ZQ analyzed the data. PC drafted the manuscript. All authors contributed to the article and approved the submitted version.

## Funding

This study was funded by Changzhou Sci & Tech Program (Grant number: CZ20220025), Changzhou High-Level Medical Talents Training Project (Grant number: 2022CZBJ069), “333 Project” of Jiangsu Province (Grant number: BRA2020157), 333 High-Level Talent Training Project (Grant number: 2022-2), “Six One Project,” Research Projects of High-level Medical Personnel of Jiangsu Province (Grant number: LGY2019025), High-level Talent Selection and Training Project of the 16th Batch of “Six Talent Peak” in Jiangsu Province (Grant number: WSN-245), Medical Scientific Research Foundation of Jiangsu Commission of Health (Grant number: H2018083).

## Conflict of interest

The authors declare that the research was conducted in the absence of any commercial or financial relationships that could be construed as a potential conflict of interest.

## Publisher’s note

All claims expressed in this article are solely those of the authors and do not necessarily represent those of their affiliated organizations, or those of the publisher, the editors and the reviewers. Any product that may be evaluated in this article, or claim that may be made by its manufacturer, is not guaranteed or endorsed by the publisher.
