# Supplementary figures and images for: MDFI is a novel biomarker for poor prognosis in LUAD
Source: Front Oncol. 2022 Oct 10;12:1005962. doi: 10.3389/fonc.2022.1005962 (PMC9589366; doi:10.3389/fonc.2022.1005962)

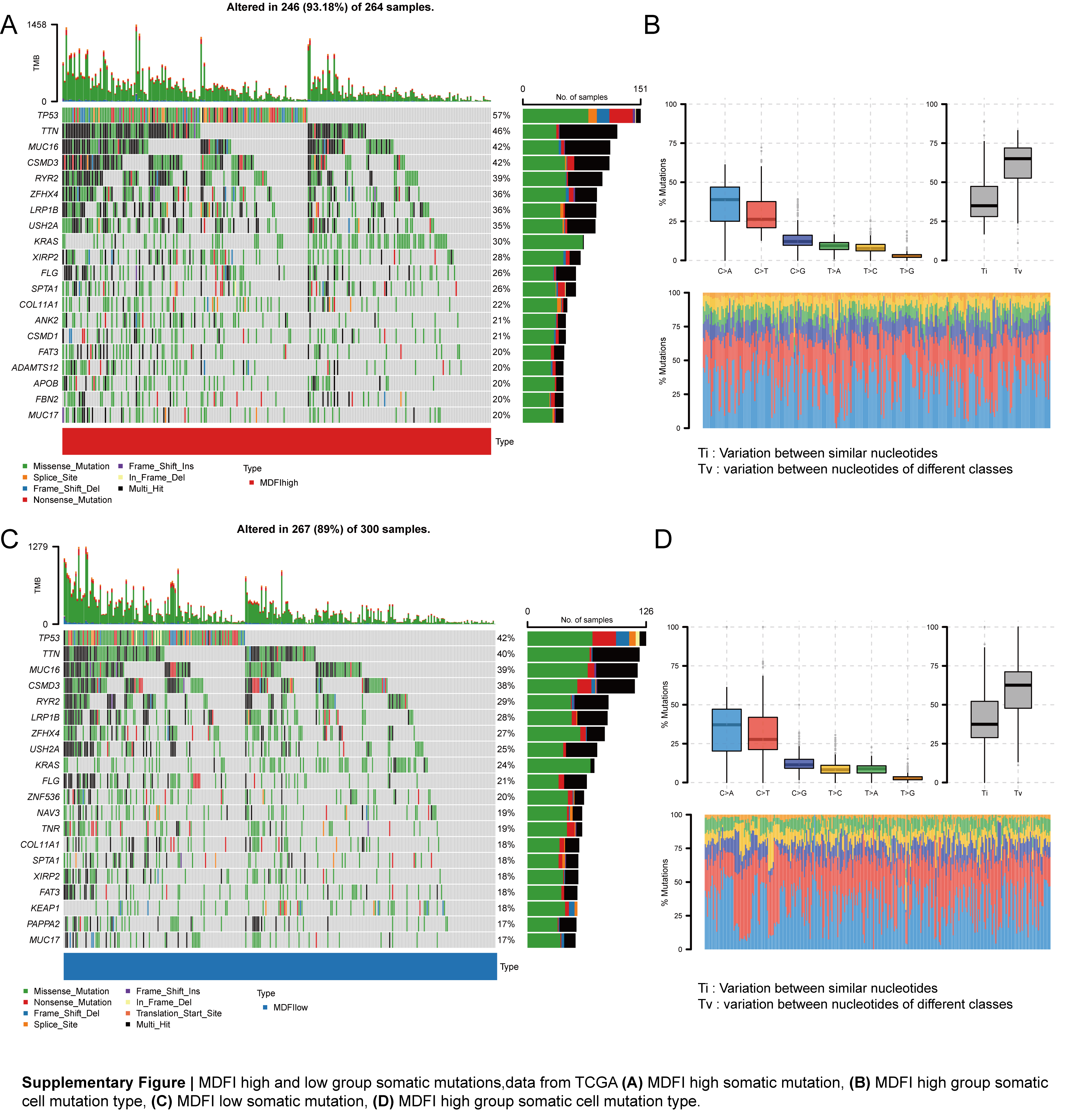

Supplement: Supplementary file 1 [file Image_1.tif]
