# Supplementary material for: MDFI is a novel biomarker for poor prognosis in LUAD
Source: Front Oncol. 2022 Oct 10;12:1005962. doi: 10.3389/fonc.2022.1005962 (PMC9589366; doi:10.3389/fonc.2022.1005962)
Supplement: Supplementary file 2 [file Table_1.docx]

**Supplementary Table.** Baseline table of 140 NSCLC patients

| Characteristic | levels | Overall (%) |
| --- | --- | --- |
| Age (years), | ⩽60 | 66 (47.1%) |
|  | >60 | 74 (52.9%) |
| Gender | male | 112 (80%) |
|  | female | 28 (20%) |
| Location | Left | 64 (45.7%) |
|  | Right | 76 (54.3%) |
| Pathologic type | Squamous cell carcinoma | 80 (57.1%) |
|  | Adenocarcinoma | 51 (36.4%) |
|  | Adenosquamous cell carcinoma | 5 (3.6%) |
|  | Sarcomatoid carcinoma | 1 (0.7%) |
|  | Bronchioloalveolar carcinoma | 3 (2.1%) |
| T stage | 1 | 15 (10.7%) |
|  | 2 | 105 (75%) |
|  | 3 | 20 (14.3%) |
| N stage | 0 | 82 (58.6%) |
|  | 1 | 31 (22.1%) |
|  | 2 | 27(19.3%) |
| M stage | 0 | 56 (40%) |
|  | 1 | 84 (60%) |
| NSCLC stage | I | 67 (47.9%) |
|  | II | 44 (31.4%) |
|  | III | 29 (20.7%) |
